# Insight into treatment of HIV infection from viral dynamics models

**DOI:** 10.1111/imr.12698

**Published:** 2018-08-11

**Authors:** Alison L. Hill, Daniel I. S. Rosenbloom, Martin A. Nowak, Robert F. Siliciano

**Affiliations:** ^1^ Program for Evolutionary Dynamics Harvard University Cambridge Massachusetts; ^2^ Department of Pharmacokinetics Pharmacodynamics, & Drug Metabolism Merck Research Laboratories Kenilworth New Jersey; ^3^ Department of Medicine Johns Hopkins University School of Medicine Baltimore Maryland; ^4^ Howard Hughes Medical Institute Baltimore Maryland

**Keywords:** antiretroviral therapy, cure, HIV, pharmacodynamics, viral dynamics model, viral rebound

## Abstract

The odds of living a long and healthy life with HIV infection have dramatically improved with the advent of combination antiretroviral therapy. Along with the early development and clinical trials of these drugs, and new field of research emerged called *viral dynamics*, which uses mathematical models to interpret and predict the time‐course of viral levels during infection and how they are altered by treatment. In this review, we summarize the contributions that virus dynamics models have made to understanding the pathophysiology of infection and to designing effective therapies. This includes studies of the multiphasic decay of viral load when antiretroviral therapy is given, the evolution of drug resistance, the long‐term persistence latently infected cells, and the rebound of viremia when drugs are stopped. We additionally discuss new work applying viral dynamics models to new classes of investigational treatment for HIV, including latency‐reversing agents and immunotherapy.

## 1 INTRODUCTION

HIV, the causative agent of AIDS, infects nearly 40 million people worldwide^1^ and represents one of the highest overall global burdens of disease.^2^ After an estimated entry into the human population in the early 20th century,^3^ it spread unnoticed until 1981 when a syndrome of opportunistic infections in previously healthy gay men^4^ led to the eventual characterization of the disease AIDS and identification of the virus responsible.^5^ Since then, HIV has become one of the most intensively studied infections. These studies have addressed how it leads to massive reductions in CD4+ T‐cell populations due to a combination of direct infection and generalized dysregulation of the immune system, and how it evolves rapidly to evade antiviral immune defenses. Massive drug development efforts starting soon after identification of the virus have resulted in 27 different approved antiretroviral drugs,^6^ which can halt viral replication and prevent transmission and progression to AIDS. Yet despite these advances, we still do not have a clear explanation for the pathogenesis of infection nor therapies that can permanently cure the infection.

**Figure 1:**
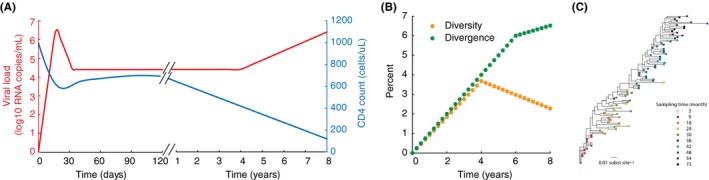
Some features of the natural history of HIV infection. (A) Approximate time‐course of HIV infection, with estimates of kinetics of viral load and CD4 count taken from longitudinal studies of acute or chronic infection.^8^, ^10^, ^13^ (B) Typical time‐course of diversity (average pairwise distance between sequences) and divergence (percent genetic distance from first detected virus) of viral populations over the course of infection, with rates taken from Shankarappa et al.^10^ (C) Maximum likelihood phylogenetic tree for viral sequences sampled over 6 years of infection (image taken from^14^ under CCBY license, created with data from Patient 6 in^10^).

The rapid pace of HIV research has been made possible by two very convenient aspects of the infection: first, the CD4+ T cells that the virus infects continually circulate through the body, allowing them to be sampled in the peripheral blood, and second, virions are released by infected cells in sufficient amounts that they can also easily be measured in blood samples and tracked over time. Genomic viral RNA in virions can be readily quantified with RT‐PCR, providing a convenient, reliable, and quantitative biomarker of infection status. Measurement of plasma HIV RNA (often referred to as “viral load”) led to the observation of complex yet repeatable patterns in individual patients over time (Figure 1). After initial infection (through sexual transmission, contaminated blood products, intravenous drug use, or perinatal events), viral levels increase exponentially with a doubling time of ~.65 days,^7^ reaching peak “viral loads” as high as 10^8^ copies of viral RNA per mL of plasma (c/mL).^8^ Viral loads then decrease over the period of a few weeks to a “setpoint” typically between 10^3^ and 10^6^ c/mL, where they can remain relatively stable for many years.^9^ During this time, viral populations diversify and diverge from the strains that founded infection,^10^ often displaying population genetic signs of strong selection.^11^, ^12^ CD4+ T cells slowly decrease over the course of chronic infection and eventually become so low (<200 cells/uL blood) that opportunistic infections occur and the individual classified as having AIDS. Early in the epidemic, these characteristic trends inspired the use of mathematical models to understand these dynamics and help generate ideas about how to treat the infection.

Mathematical models are sets of equations or rules that describe how different entities in a system interact and change over time.^15^ Different models may consider dynamics at very different scales — from individual molecules to cells to people to countries. Most commonly, models are formulated as systems of nonlinear differential equations or as sets of stochastic reactions constituting a Markov process. Roughly speaking, the use of models in biology can be divided into two cases. In one scenario, models may be constructed with the goal of explaining patterns that are observed in existing data, perhaps for generating and comparing hypotheses about the mechanisms that lead to the observed data or to estimate values of particular model parameters. While this approach has the advantage of allowing direct comparison of models with data, it has the downside that it is generally always possible to create a model that reproduces observed data, but this does not mean that model is correct or useful. Alternatively, models may be constructed in the absence of directly related data, by starting from a basic mechanistic understanding of the biological processes involved and choosing only the processes considered most critical to the outcome. Values for reaction rates can ideally be taken from direct measurement of individual steps in the process. Constructing such a model is a formal way of integrating often disparate data into a single framework, and can be used to predict the outcomes of studies that have not yet been conducted based on the optimal use of prior information. Ideally, models can be developed and refined by iterating between these two approaches.

In this paper, we will review some examples of how mathematical models have improved our understanding of HIV treatment, including both successes and failures. The models we will discuss are commonly called “viral dynamics” models and track levels of virus and immune cells over time within individual infected people or animals (and thus are often referred to as “within‐host” models). A huge amount of other work that will not be discussed here uses “between‐host” models to describe how HIV spreads between individuals in a population (eg, References 16, 17, 18). The first half of the paper will focus on antiretroviral drugs, which are still the only approved drugs for treating HIV. The second half of the paper will discuss investigational therapies being tested with the hope that they may one day replace combination antiretroviral therapy (ART) by permanently curing the infection. Many other excellent reviews of viral dynamic modeling of HIV exist in the literature (eg, References 19, 20, 21). Here, we do not attempt to cover the entire field but rather to detail some topics we personally have studied or feel are illustrative examples of these methods.

### 1.1 Basic viral dynamics model

**Figure 2:**
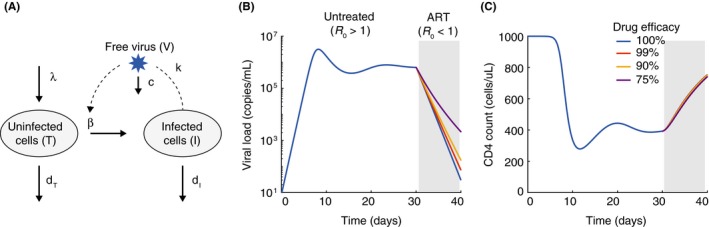
The basic viral dynamics model for HIV. (A) Diagram of the variables and reactions tracked by the model, as described in the text. B‐C) Example time‐course of viral loads (B) and CD4 counts (C) from the model, starting from initial infection, for 30 days before 10 days of antiretroviral therapy. We assume that therapy changes *β*. As long as therapy leads to *R*_0_ < 1, the decay slope is not very sensitive to the treatment efficacy. Parameters for the model were: *λ* = 100 cells/uL/d, *β *= 3× 10^−7^/(virus/mL)/d, *d*_*T*_ = .1/d, *d*_*I*_ = 1/d, *k *=* *250 virus/cell/d, *c *=* *25/d. For these parameter values *R*_0_ = 3. With treatment, where is the treatment efficacy. The initial condition for was *T*(0) = *λ* /*d*_*T*_,* I*(0) = 10^−3^ cell/uL/d, *V*(0) = 0.

The backbone of most mathematical modeling work describing HIV infection within individual patients is the basic viral dynamics model. In its simplest form, this model tracks levels of the virus (*V*) and the CD4+ T cells that it infects (Figure 2a). Uninfected target cells (*T*) are assumed to enter the system at a constant rate *λ*, and die with rate constant *d*_*T*_ (equivalent to an average lifespan of 1/*d*_*T*_). Infected cells (*I*) are produced with a rate proportion to levels of both virus and target cells and the infectivity parameter *β*. Infected cells produce free virus at a rate *k* and die with rate *d*_*I*_ (which is assumed to be higher than *d*_*T*_). Free virus is cleared at rate *c*. These reactions can be described with the set of ordinary differential equations.

$$\dot{T}=\lambda -\beta TV-d_{T}T$$

$$\dot{I}=\beta TV-d_{I}I$$

(1) $$\dot{V}=kI-cV$$

This model reproduces many of the qualitative features of acute and chronic HIV infection (Figure 2B,C). Following transmission of a small number of founder virions, viral loads initially grow exponentially, then peak before declining to eventually reach a stable setpoint level. Formulas can be derived from the model relating these features of viral load to the underlying parameter values.^19^ An important early insight provided by this model was that the decline from peak viremia could be explained without any specific later‐onset immune response. It is a natural consequence of the slower turnover of uninfected cells relative to infected ones.^22^ Because of the nonlinear infection rate that appears in Equation [Link], the model displays a type of thresholding behavior in which the infection can only spread and persist if a parameter combination called the “basic reproductive number” is large enough. Otherwise, the virus will be cleared from the body. The basic reproductive number is given by the formula^19^, ^23^

(2) $$R_{0}=\frac{\lambda \beta k}{d_{T}d_{I}c}$$

Mathematically, *R*_0_ = 1 is a “transcritical bifurcation” in the system, and only for *R*_0_ > 1 is the extent of infection nonzero in the long run. *R*_0_ has an intuitive meaning: It is the average number of secondary infections caused by virions released from a single infected cell over its lifetime in a population of otherwise uninfected target cells. This phenomenon is exactly analogous to the concept of the basic reproductive number in population‐level epidemic models,^24^ with people or animals replaced by cells. The goal of treatment for any infection, in an individual or population, is to alter one of the parameter values such that *R*_0_ < 1.

The rate parameters for this model can be estimated in a number of different ways. Lifespans of immune cells can be estimated from in vivo heavy‐water labeling experiments,^25^ and clearance rates of virus have been estimated from plasma apheresis.^26^ Before infection, CD4+ T cell levels are stable at measurable levels, and once the death rate is known, the production rate can be estimated as the quantity needed to achieve an equilibrium. In vitro infections, measurements of infected cell and virus levels in vivo, and ex vivo tissue imaging studies have been used to estimate the number of virions produced per infected cell.^27^ While it is generally impossible to measure the infection rate *β* directly, the observed rate of exponential increase or the value of the viral load setpoint can be used to estimate it when other parameters are known. Another technique is to attempt to jointly estimate all parameters by fitting the model to longitudinal data, though in general all parameters of the model are not uniquely identifiable this way.^28^ Estimates of the basic reproductive number suggest values anywhere between 2 and 25 (median ~8),^7^ implying that treatments must inhibit at least 95% of infections to lead to clearance (need, where is the treatment efficacy).

## 2 MODELING ANTIRETROVIRAL THERAPY

### 2.1 What does viral load decay during ART tell us about the underlying dynamics of infection?

Perhaps the most influential use of this model has been in interpreting changes in viral load when antiretroviral drugs are administered.^29^, ^30^ When ART is given, the viral load almost immediately begins to decay exponentially with a slope of ~1/d. Combination ART prevents successful infection of new cells, and if therapy is 100% effective, *β* → 0 and the viral dynamics equations have an analytic solution for viral load over time.^29^

(3) $$V\left(t\right)=V_{0}\frac{\left(ce^{-d_{I}t}-d_{I}e^{-ct}\right)}{c-d_{I}}$$

where *V*_0_ is the viral load at the time of therapy initiation. Thus, the decay dynamics only depend on the lifespans of free virus and infected cells: viral load will decay with the slower of these two values after a shoulder phase approximately equal to the length of shorter lifespan. Since the lifespan of free virus is estimated to be around *1/c* ~ 1 h,^26^ but the observed decay rate is around 1/d, we must have *d*_*I*_ *> c* and *d*_*I*_ ~ 1/d.

When this decay was first observed and interpreted in the context of this model,^29^, ^30^, ^31^ it was very surprising that virus‐producing cells had such a short lifespan. This lifespan implies that many new cells must be infected each day to maintain setpoint viremia (estimates of *d*_*I*_ *I* at setpoint from the model in Figure 2 scaled up to full body cell numbers are around 10^9^). This meant that the long period of asymptomatic infection and constant viral levels prior to the development of AIDS was not due to a latent or slow moving infection, but instead a highly dynamic balance between new rounds of infection and the death of infected cells. Since HIV generates a new mutation approximately every three infection events (mutation rate per base pair per replication cycle of 3× 10^−5^ and genome length of ~10 kb^19^), these numbers allow for a tremendous amount of diversity to be generated, explaining the rapid rates of evolution observed.

Despite these and many other insights into HIV infection that have come from the viral dynamics model, it is important to note that the model does make a number of unrealistic assumptions. For example, this model assumes that cells start producing virus immediately upon being infected, whereas in reality a cell must pass through multiple stages of the viral lifecycle before infectious virions are released. Additions to this model include this time delay,^32^, ^33^, ^34^ which has many interesting effects, but most importantly, changes the relationship between the early viral growth rate and estimates of *R*_0_.^7^ CD4+ T cells obey very simplified dynamics in these equations, but are actually governed by more complicated homeostatic mechanisms that increase cell proliferation when numbers get low.^35^, ^36^ While CD4 +  T cell levels can decline dramatically during chronic infection, generally only activated cells are highly susceptible to infection, and only a very small fraction of them are infected at any given time (around 1/1000).^37^, ^38^ Including more of these details can improve the agreement between model predictions and observed CD4 counts but still cannot explain the entire progression to AIDS.^39^

Infected cells and free virus are not generally cleared at a constant rate throughout infection because they are targeted and cleared by adaptive immune responses that expand in response to infection. Many models of antiviral immunity have been developed to explain different features of infection.^12^, ^19^, ^40^, ^41^ Inclusion of immune system effects is needed to reproduce the large drops from peak viremia to setpoint^42^, ^43^ and explain patterns of viral evolution (eg, References 40, 44, 45). When treatment reduces *R*_0_ < 1 in this model, the simplest forms of the model predict that infection will eventually be completely cleared. However, early studies demonstrated that no matter how long antiretroviral therapy is given and plasma viral levels remain undetectable by standard clinical assays, the infection always returns once therapy is stopped.^46^, ^47^ This was found to be due to the presence of a “latent reservoir” of integrated proviral genomes in resting memory CD4+ T. These latent genomes are not transcribed into mRNA and translated in protein to complete the viral lifecycle due to the quiescent state of these cells.^48^ However, upon cellular activation, transcription and translation can resume. Latently infected cells can persist despite decades of therapy,^49^, ^50^ and reactivate later to restart infection.^51^, ^52^, ^53^ Consequently, antiretroviral therapy is not curative and currently must be taken for life. Models that include viral latency are now common in studies of both antiretroviral therapy and new curative strategies (Reference 54 and discussed in later section).

Interestingly, many of these more complicated facets of infection can actually be inferred from looking more closely at viral load decay curves under different types of treatments. For example, after the first weeks of treatment with combination ART, the rate of viral load decay slows down, from a half‐life of less than a day to a half‐life of around 2 weeks. Then, after 3–6 months of treatment, the viral load becomes undetectable by standard clinical assays (limit of detection 50 c/mL), but ultrasensitive assays can reveal continual low‐level viremia which decays extremely slowly, if at all (Figure 1B). Mathematical models have been used to interpret this decay, and in general the multiple phases are believed to reflect distinct populations of infected cells (eg, Reference 55, 56, 57, 58). The final phase of decay is now understood to be release of virus following reactivation of cells from long‐lived latent reservoirs, and the decay reflects the very slow decline in the number of latently infected cells.^49^, ^59^ The identity of the cells responsible for the second phase of decay is not yet clear. Another cell type that HIV can infect, macrophages, was suspected, but has now been ruled out,^60^ while other possibilities include partially resting CD4+ T cells in a lower state of activation, cells with a type of preintegration latency, release of virus from follicular dendritic cells, or simply a decreased death rate of actively infected cells due to waning immunity.^58^, ^61^, ^62^

**Figure 3:**
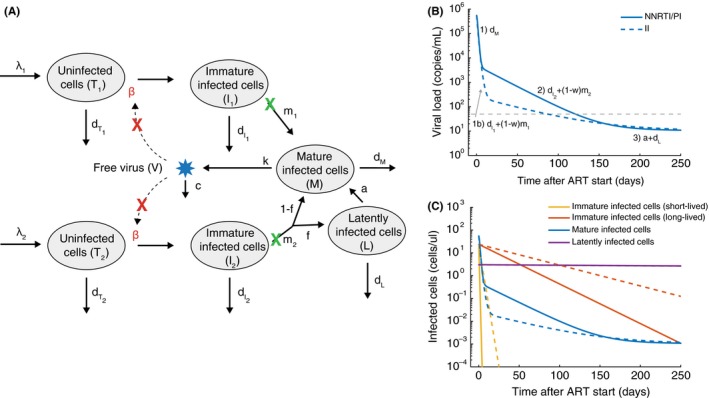
Multiphasic viral load decay under antiretroviral therapy suggests more complex infection dynamics. (A) A schematic of an augmented viral dynamics model that has emerged from various studies of viral load dynamics during therapy.^55^, ^56^, ^57^, ^58^, ^59^, ^66^, ^68^ Most drugs inhibit the ability of virus to infect new cells (red X). Two separate populations of target cells are hypothesized to exist, with the second type (*T*_*2*_ *, I*_*2*_) being longer lived and proceeding to integrate virus more slowly. Infected cells of either type can be divided up into those who have not yet completed the phase of the viral lifecycle where integration occurs (“Immature”, *I*_*i*_), and those who have (“Mature”, *M*), which allows investigation of different dynamics in the presence and absence of integrase inhibitor drugs (green X). Some infected cells transition to an extremely long‐lived latent state (L). (B) Viral load decay with and without integrase inhibitors (II) (commonly used alternative drugs are protease inhibitors (PI) and nonnucleoside reverse‐transcriptase inhibitors (NNRTI)). Labels above phases of decay show the parameter combinations responsible for each phase. (C) Dynamics of the different infected cell combinations predicted by the model. Dotted vs dashed lines have same meaning as in B. The model was created by combining conclusions from various papers. Values for parameters are also taken approximately from these studies: *λ*_1_ = 100 cells/uL/d, *β *= 3× 10^−7^/(virus/mL)/d, $d_{T_{1}}=.1/d,\;d_{I_{1}}$ = .36/d, *m*_1_ = 2.6/d, *λ*_2_ = 1 cells/uL/d, $d_{T_{2}}=.1/d,\;d_{T_{2}}$ = .02/d, *m*_1_ = .02/d, *f* = 10^−2^, *a* = 4× 10^−4^/d, *d*_*L*_ = 10^−4^/d, *d*_*m*_= 1/d, *k *=* *250 virus/cell/d, *c *=* *25/d. With treatment with NNRTI or PI,* β* → 0, and for treatment with II,* m* → *m*(1 − ω) and the treatment efficacy where ω = .95.

Further insight has been gained by comparing viral load decay curves in treatment with and without the integrase inhibitor (II) class of drug. Early on after this class was introduced, it was noticed that viral loads became suppressed faster than with reverse‐transcriptase (RTI) or protease inhibitor therapy (PI). This was initially taken as evidence that these drugs were more efficacious, but for the reasons detailed above (Figure 2, lack of dependence of decay curves on drug efficacy), modelers cautioned against this interpretation and hypothesized that the altered kinetics may be due to the later stage in the lifecycle at which the integrase inhibitor class acts.^63^, ^64^, ^65^ Recent work by Cardozo et al.^58^ used densely longitudinally sampled viral load data^66^ during treatment with either (a) 3 RTIs + 1 PI, (b) 1 II, or (c) 2 RTIs + 1 II to compare various models to fit the decay curves. Based on the various alterations in kinetics seen with the II (first phase viral decay separating into two phases, (1a) and (1b), second phase decay occurring later and slower), they identified the model that fit the data best without unnecessary complexity. They concluded that the virus infects two distinct cell subsets, one with a fast rate of integration and another with a slow rate of integration, but that once integration occurs, production of virions occurs with similar rates in each subset. Additionally, their results suggest that the decay curves can only be explained if integrase inhibitors are not 100% effective even at the high concentrations administered, so that some integration proceeds slowly even in the presence of the drug. This agrees with direct measurements of drug efficacy in ex vivo assays (discussed in next section),^67^ and could be due to the ability of HIV genes to be expressed at low levels from unintegrated viral DNA. In Figure 3, we show the infection model that has emerged from these combined studies and the decay curves that are produced under different treatment regimes.

### 2.2 How efficacious are antiretroviral drugs?

HIV drugs rapidly reduce viral loads, but they do not eliminate all of the problems associated with HIV infection. Older regimes included drugs with unpleasant or even toxic side effects, making it difficult for patients to consistently adhere to therapy, or necessitating treatment changes. If there are problems with adherence, the drugs can fail to keep viral load suppressed indefinitely, often due to the development of drug‐resistant virus that can replicate despite the presence of therapy. Treatment success depends critically on choosing a drug dose that minimizes adverse effects while preserving efficacy, and on choosing drug combinations that inhibit viral replication and slow down the evolution of drug resistance. Since the number of possible drug combinations and doses is simply too high to test each in clinical trials, it is imperative to have models for drug efficacy that can be used to make informed decisions about administering therapy. In particular, these predictions require an understanding of the relationship between the concentration of a drug in the bloodstream and the reduction in the new infection events.

**Figure 4:**
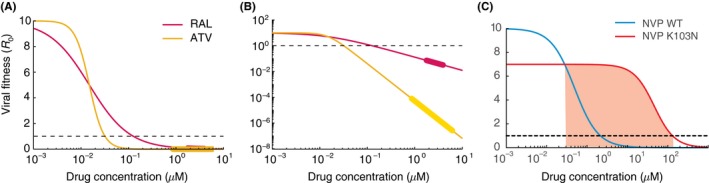
Effects of antiretroviral drugs on viral infectivity. (A) Dose‐response curves measured in single‐round ex vivo assays for the integrase inhibitor raltegravir (RAL,* IC*_50_ = .015 uM,* m* = 1) and the protease inhibitor atazanavir (ATV,* IC*_50_ = .015 uM, *m* = 2.9). The assay measures *f*_*u*_ (Equation [Link]), the fraction of uninhibited infections compared to the absence of drug, and we scale this up to effective basic reproductive number by multiplying by drug‐free *R*_0_ = 10. Below *R*_0_ = 1 infection be controlled. (B) Same as A but plotted on log‐log scale to highlight differences in suppression between drugs at clinically relevant concentrations. The thicker regions on the lines are drug levels between the typical peak and trough concentrations when drug is taken daily with perfect adherence. (C) Dose‐response curves for wildtype (blue) and the K103N mutant (red) for the NNRTI nevirapine (NVP). Red shaded area is the “mutant selection window”, the range of drug concentrations where a resistant strain could outcompete wildtype and cause treatment failure. Parameters taken from.^67^, ^69^

The previous sections emphasized that the slope of viral load decay during therapy reflects of an important timescale in infection: the lifespan of virus‐producing cells. However, analyzing these curves tells us very little about drug efficacy itself; that is, what percent of new infections are blocked in the presence of drug? Viral dynamics models show that as long as therapy reduces viral replication below a critical threshold needed to push infection toward elimination (*R*_0_ < 1), the slope of viral load decay is relatively insensitive to the exact drug efficacy (Figure 2B). For example, drugs that stop 90% vs 99% vs 99.99% of new infections when *R*_0_ ~ 4 are nearly impossible to distinguish. However, the differences in residual replication under these different hypothetical regimens could be very important in determining the risk of developing resistance, or the risk of losing suppression if a few drug doses are missed. Even if viral load kinetics were more sensitive to drug inhibition, it would still be difficult to use it to reconstruct a dose‐response curve for drug efficacy vs concentration, since drug levels fluctuate significantly between doses due to the physiologic processes of absorption, distribution, metabolism, and elimination (Figure 4).

In vitro assays, which allow virus to spread in cell culture systems with fixed drug levels, can avoid these problems, but their physiological relevance could be minimal, since cell lines in culture media may respond to infection and treatment very differently than cells in vivo. Additionally, the relationship between the amount of infection after a certain time of spreading in culture and the actual reduction in per contact probability of infection could be highly nonlinear and dependent on other parameters in the system which are hard to control. Many of these problems can be overcome with a unique infection assay introduced by the Siliciano laboratory^70^ and responsible for the most quantitative description of antiretroviral efficacy that we currently have. The assay is conducted in primary CD4+ T cells isolated from healthy donors and cultured in human blood serum, and uses virus that is capable of only a single round of infection and that labels productively infected cells by expressing a fluorescent protein along with viral genes. By comparing the fraction of cells infected in the presence vs absence of drug using flow cytometry, the inhibition of the drug can be quantified over many orders of magnitude.

Using this assay, Shen et al.^67^ generated dose‐response curves describing the relationship between drug concentration (*D*) and the fraction of infections inhibited (compared to the absence of drug) for all approved antiretroviral drugs. These curves could be described extremely well by a simple two‐parameter “Hill”‐function^71^:

(4) $$f_{u}=1-\in =\frac{1}{1+{\left(\frac{D}{IC_{50}}\right)}^{m}}$$

Here, *f*_*u*_ is the fraction of infections that are *unaffected* by the drug (equivalent to one minus the drug efficacy). The *IC*_50_ describes the concentration of drug that reduces infections to one half the drug‐free level, while the slope, *m*, describes how quickly inhibition changes as drug levels move away from the *IC*_50._

Drugs differ substantially in their *IC*_50_ values, and there is overall no relationship between the drug class (determined by the phase of the viral lifecycle the drug inhibits) and the *IC*_50_. In contrast, striking differences between drug classes were observed in the slope of the dose‐response curves (*m*). Nucleoside reverse‐transcriptase inhibitors (NRTIs) and integrase inhibitors (IIs) all had slopes very close to 1, while values were near 1.7 for nonnucleoside reverse‐transcriptase inhibitors (NNRTIs) and fusion inhibitors (FI). Slopes for protease inhibitors ranged from ~2 to ~4.5 (Figure 4).

These results were surprising for a few reasons. First, HIV drug efficacy (as measured by older assays), was previously only reported in terms of the *IC*_50_, but inhibition at the higher concentrations which are required clinically is highly dependent on the slope as well (Figure 4). The total viral inhibition at clinical drug levels calculated from these assays is higher in drug combinations recommended for first‐line treatment and in those that outperform others in head‐to‐head randomized clinical trials.^67^ Second, the functional form for the Hill curve comes from considering a general mechanistic model of enzyme‐substrate kinetics, and in this model the slope is directly related to cooperatively, which in this case would describe how many drug molecules must be bound to a target to inhibit it.^72^ However, for HIV drugs of most classes, there is only a single drug‐binding site on each target, making slope values >1 very puzzling. A solution to this dilemma was proposed by in a follow‐up paper by Shen et al.,^67^ using what they call a “critical subset model”.^73^ They pointed out that for some targets of HIV drugs, there are multiple copies of the target in a single virion or infected cell. Although they are not covalently linked, these target molecules work together to carry out the relevant reactions (eg, protease cleavage of HIV polyproteins) and complete the relevant step in the virus lifecycle. Inhibition of infection may require that some critical fraction of targets be bound to the inhibitor. Consequently, the dose‐dependence of infection inhibition may act like the case of cooperative binding of a multivalent target. A mechanistic model of this kind produces dose‐response curves that look very similar to Hill curves with slopes greater than 1. Interestingly, there are other situations in which only a single drug target per infected cell is relevant. For example, the integration of viral DNA into the host cell genome or the addition of a dNTP to a growing HIV cDNA chain during reverse transcription. In these cases, the measured slope values are close to 1.

In situations where drug levels are suboptimal, viral replication can occur and drug‐resistant variants can arise.^74^ Resistance is not an all‐or‐nothing phenomenon, and most mutations only confer partial resistance. To quantify the degree of resistance, viruses can be generated in the laboratory with specific suspected drug resistance mutations, and then subjected to the same dose‐response curve measurements described above.^75^ Overall, the dose‐response curve shifts in three possible ways for each resistant strain. In the absence of drug, mutant strains tended to have lower infection rates than wildtype strains. This “cost of resistance” is well‐documented in many systems and occurs because of compromises in the function of viral proteins that occur when they undergo amino acid changes to avoid drug effects.^76^, ^77^, ^78^, ^79^, ^80^ Since this fitness cost shifts the entire dose‐response curve down (Figure 4C), it also indirectly influences how much a resistant strain can replicate at any drug level. In the presence of drug, mutant strains in general have higher *IC*_50_ values as well as lower slope values. Single point mutations to NNRTIs tend to have larger effects on both parameters overall, and resistance to integrase inhibitors only significantly alters the *IC*_50_. Interestingly, resistance to PIs tends to change only the slope. Major public databases that characterize the resistance levels of different mutations^81^ as well as commercial testing services rely heavily on the *IC*_50_ to quantify resistance, but residual replication at clinical drug levels, especially to PIs, cannot be predicted without considering the three resistance parameters together.

Modern antiretroviral therapy typically involves combinations of three drugs, which makes it unlikely that viral strains containing resistance mutations to all three drugs will preexist at the time therapy is started or emerge during treatment.^68^, ^82^, ^83^ Understanding the combined reduction in viral replication would be helpful for designing optimal drug combinations. Early work in this field of pharmacodynamics theorized that inhibition by drug combinations could be calculated depending on whether the drugs act on the same or different target molecules.^84^ Based on that idea and the known mechanisms of action of HIV drugs, Jilek et al.,^85^ studied the effect of combinations of two antiretroviral drugs and found that while some two‐drug combinations behaved as expected, others interacted in surprising ways: sometimes the overall inhibition was much higher (“synergy”) or much lower (“antagonism”) than expected. Moreover, the combined inhibitory effect of drug combinations was highly correlated with treatment outcomes in clinical trials. Characterizing these relationships for all three drug combinations is logistically impossible ((25 drugs)^3^ * (10 doses)^3^ ~ 10^7^) but Jilek et al. examined a subset, and found that three‐drug efficacy could be predicted entirely from two‐drug data, meaning that all drug‐drug pharmacodynamic interactions were pairwise. Interestingly, similar results have been obtained for *Escherichia coli* and *Staphylococcus aureus* treated with antibiotics^86^ or cancer cell lines treated with chemotherapy drugs,^87^, ^88^ despite the use of completely different biological systems, more complicated mechanisms of drug action, and different models to describe drug efficacy.

### 2.3 How does antiretroviral efficacy and adherence influence treatment outcomes?

Dose‐response curves tell us how much infection is instantaneously blocked at a given drug level, but they do not directly tell us what the long‐term outcome of treatment will be. Drug levels fluctuate over time as drug is absorbed after a dose is taken and then cleared, and the individual pill‐taking behavior of each person, including their potentially suboptimal adherence, can lead to more extreme peaks and troughs in drug levels. The pretreatment viral population size, the mutation rate, and the residual replication of wildtype virus despite therapy determine the likelihood of generating resistant strains, while the drug level and the way the mutation shifts the dose‐response curve determine the likelihood that a resistant strain is selected and grows in the body. Understanding the complex interaction between all these factors and how they combine to determine therapy outcomes is impossible without mathematical models.

**Figure 5:**
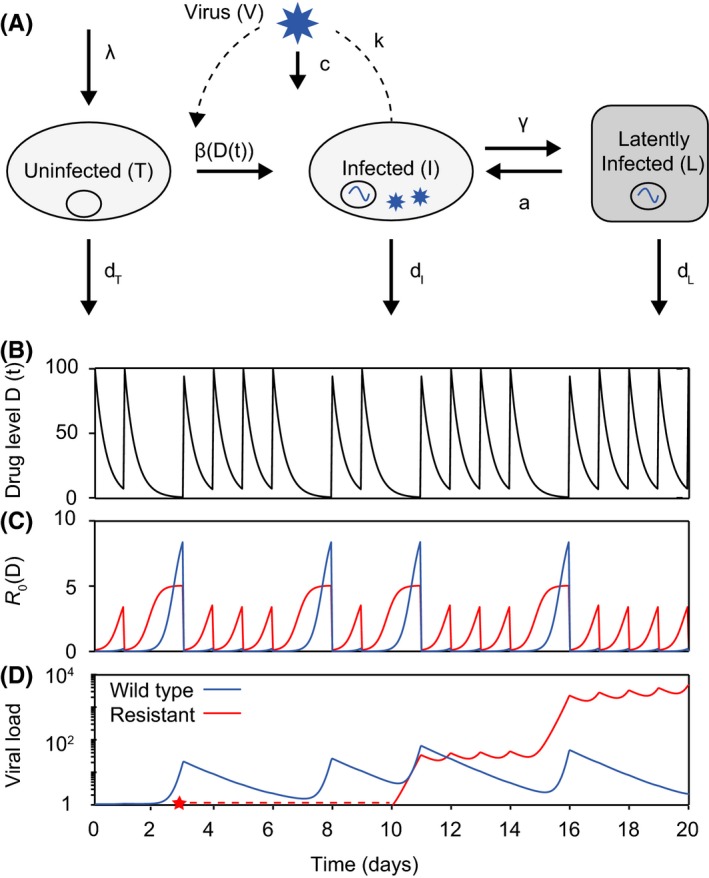
An augmented viral dynamics model can be used to simulate antiretroviral therapy and the evolution of drug resistance. (A) Basic viral dynamics model with the addition of a population of latently infected cells. A separate strain of virus, and the cells it infects, can be tracked for each genotype (*V*_*i*_ *, I*_*i*_ *, L*_*i*_). (B) Drug levels over time for a pill taken daily, and assumed to increase to maximum concentration immediately afterwards then decay exponentially. Each dose is taken with a 70% probability. (C) Basic reproductive number over time as a function of drug levels. (D) Levels of wildtype and resistant virus over time. At time zero, there is no resistant strain, but it is produced via mutation from the wildtype at the point indicated by the red star. Drug parameters: *IC*_50_ = 1, *C*_*max*_ = 100, *m *=* *1, half‐life=6 hours. The resistant mutant has a 10‐fold increase in *IC*_50_ and a twofold decrease in baseline fitness. Baseline *R*_0_ = 10.

The viral dynamics models described in the first section can be modified to consider drug treatment in more detail.^69^, ^89^, ^90^, ^91^, ^92^ Although different drug classes act on different stages of the viral lifecycle (which are not explicitly considered in the basic viral dynamics model), their effects can be approximated by a dose‐dependent reduction in the rate at which virus infects cells (*β* → *β* (*D*), where *β* (*D*) is simply the drug‐free value *β*_0_ multiplied by the drug effect (Equation [Link]). Since drug concentrations are time‐dependent, *D* can be replaced by *D*(*t*). Therefore, viral population dynamics proceed under a time‐dependent infection rate (Figure 5).

To include the possibility of the evolution of drug resistance, the basic model can be augmented to include multiple strains of virus (wildtype/drug‐susceptible, mutant/drug‐resistant) which compete for target cells and can be produced by other strains via mutation (Figure 5A). It is important that models for long‐term therapy outcomes include a compartment of latently infected cells, which are seeded by active infection and can reactivate to produce actively infected cells, since otherwise temporary admistration of fully suppressive therapy would falsely be predicted to cure infection. Overall, this results in a multistrain, multicell type model with time‐dependent parameters (Figure 5D), which can be described by the following set of equations:

$$\dot{T}=\lambda -T\sum_{i}\beta_{i}\left(t\right)V_{i}-d_{T}T$$

$${\dot{I}}_{l}=T\sum_{j}\beta_{j}\left(t\right)V_{j}Q_{\mathrm{ij}}-\left(d_{I}+\gamma \right)I_{i}-aL_{i}$$

$${\dot{V}}_{l}=kI_{i}-cV_{i}$$

(5) $${\dot{L}}_{l}=\gamma I_{i}-\left(a+d_{L}\right)L_{i}$$

where the new variable *L* is the level of latently infected cells, and the subscript *i* refers to the genotype of the viral strain. This formulation assumes that strains differ only in their infection rates (*β*_*i*_). Actively infected cells transition into latent infection at rate γ, *a* is the rate at which latently infected cells reactivate, and *d*_*L*_ is the death rate of latently infected cells. *Q* is a matrix that includes information both on the mutation rate and the genetic structure of the population, ie, is the probability that a cell initially infected by a virion of genotype *i* ends up carrying genotype *j* due to mutation during the reverse transcription process is *Q*_*ij*_. The rates governing latently infected cells tend to be much smaller than those for activated cells or virus (eg, *d*_*L*_, γ, *a* ≪ *d*_*I*_, *d*_*T*_).

Even without dynamically simulating such a model, important insight can be gained on potential treatment outcomes just from at the relative dose‐dependence of mutant and wildtype viral fitness^69^, ^89^ (Figure 4D). An important mathematical feature of the viral dynamics model is that it displays competitive exclusion; that is, one strain will always dominate the population while the other is reduced to low levels sustained only by new mutations or release from latent reservoirs. The “winner” of such a competition is the strain with the highest *R*_0_. If all strains have *R*_0_ < 1, infection will be suppressed (reduced to a level sustained only by latent cells while they remain). Thus, when drug levels are constant, the strain with the highest beta value determines treatment outcome, since *R*_0_ is directly proportional to *β*. When drug levels are low, the fitness cost incurred by a resistant strain dominates, meaning that the wildtype strain has higher infectivity (Figure 4C, left of pink shading). Treatment can fail due to replication of wildtype virus. At slightly higher drug levels, if a resistant strain exists in the population, it will be selected and dominate the infection, causing treatment failure with resistance. However, if resistance does not arise, wildtype virus can still grow. At yet higher drug levels, wildtype virus is controlled by the drug, and failure can only occur if resistance is present (Figure 4C, pink shading). At very high drug levels (which may or may not be clinically achievable without toxicity), both resistant and wildtype strains will be suppressed and infection will be controlled (Figure 4C, right of pink shading).

When drug levels fluctuate due to intrinsic pharmacokinetics and adherence patterns, drug levels can oscillate between different selection regimes (Figure 5). Treatment outcomes under time‐dependent drug levels can be approximated by comparing time‐averaged *R*_0_ values between wildtype and resistant strains^89^ or by considering the fraction of time spent in each of the “selection windows”^69^ (Figure 4C). These proxies are significantly better than simply measuring time‐averaged drug concentration, which misses the highly nonlinear relationship between drug levels and viral fitness. However, they still have limited predictive power, since they ignore the fact that resistant strains do not always exist but instead must be generated stochastically via mutation before being available to be selected, and can go extinct if outcompeted temporarily.^69^ Consequently, the specific time‐course of drug levels can influence outcomes.

More predictive models of viral dynamics under drug treatment can be created by (a) moving from differential equations, which assume populations can be arbitrarily small and all processes occur continuously, to stochastic models of finite‐sized population, and (b) including realistic parameters for drug levels over time and drug effects on different viral genotypes (Figure 5). Our HIV model incorporating the experimental measurements of drug efficacy described above, the identity of the most common resistant strains and the rates at which they are generated by mutation, drug levels measured from clinical trials, and a calibrated model of viral dynamics was used to examine the relationship between patient adherence and treatment outcomes for a panel of antiretroviral drugs.^69^ We found that multiple clinically observed trends could be explained by the model, and understood based on the underlying mechanisms. For example, there is a large range of low to moderate adherence levels where NNRTI‐based therapy is prone to resistance, due to the long half‐lives of these drugs and the shallow dose‐response curves. In contrast, boosted protease inhibitor therapy is more likely to fail just due to the growth of wildtype virus. The short half‐life and sharp dose‐response curves for these drugs make the time spent in the selection window where resistant strains are favored small. Similar models have now been developed for many different infections, including HCV,^93^, ^94^ HSV,^95^ TB,^96^ and others have applied similar models to HIV^97^, ^98^, ^99^, ^100^ to examine effects such as archiving of transmitted resistant strains in the latent reservoir, tissue compartments with lower drug concentrations, details of intracellular pharmacokinetics of drugs, and host immunity.

Beyond the overall adherence level, more detailed characteristics of the drug time‐course can influence treatment outcomes. Wahl and Nowak^89^ showed that resistant strains are more likely to flourish when drug doses are taken more evenly as opposed to in a more “clumped” fashion, even when the total fraction of doses taken is the same (assuming that resistant strains always exist). When drugs are given in combination, the overlap between missed doses, which can differ depending on whether the drugs are packaged together in a “combo‐pill” or allowed to be taken separately, can determine whether or not a drug combination is “resistance‐proof”.^69^ Long‐acting therapy, which is taken much less frequently than current daily dosing due to extended half‐life formulations, is currently under development,^101^ and there are worries it may be more prone to resistance development in the presence of missed doses. Models can be used to explore this possibility, and for preliminary investigation of a once‐weekly formulation of the drugs dolutegravir and raltegravir, and suggest failure rates should be similar to daily pills with similar average drug concentrations.^102^ The periodic highs and lows of drug levels during regular therapy can also promote resistance in an unexpected way. For example, viral populations may be able to evolve the ability to “synchronize” their lifecycle with the drug period, so that they only undergo a particular lifecycle stage when drug level blocking it is at their lowest, and therefore avoid the drug effect.^34^ Whether this effect is responsible for any clinical resistance patterns for HIV is still unknown.

## 3 MODELING NOVEL THERAPIES TO PERTURB LATENT INFECTION OR BOOST IMMUNE RESPONSES

**Figure 6:**
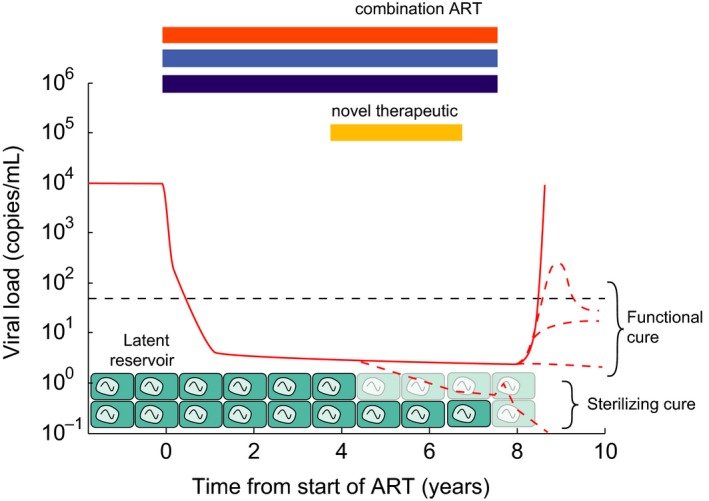
Schematic of the barriers to HIV cure and conceptual approaches to cure. Combination ART rapidly suppresses viral loads (solid red) to below clinical detection limits, but low‐level viremia released from long‐lived latently infected cells continues. Whenever therapy is stopped, viral load rebounds (solid red). “Sterilizing cure” approaches aim to reduce or completely clear the latent reservoir, or render cells in it incapable of reactivating (possible infection scenario shown in bottom red dotted line). “Functional cure” approaches aim to equip the body with the ability to control reactivating infection before full‐blown rebound occurs (effectively by reducing *R*_0_ **< 1) (three possible control scenarios shown in red dotted lines).

Antiretroviral drugs are currently the only therapies approved form of treatment specifically targeting HIV, and they have tremendous potential to control the global epidemic. Currently, approximately 18 million of the 39 million estimated HIV+ individuals are receiving combination ART,^103^ a tremendous feat of basic science, clinical medicine, public health, and political will. However, antiretroviral therapy is not curative, and must be taken daily, for life, to keep viral levels suppressed. Over the past decade, an ambitious new research agenda has developed for HIV, with the goal of finding therapies that can permanently cure the infection.^104^ There are two basic ideas for how this could be accomplished. One approach, often called a “sterilizing cure”, is to purge the body of enough residual latently infected cells that the chance that infection will be rekindled when treatment is stopped is extremely low. Another approach, often called a “functional cure”, is to equip the body with the ability to control the infection, rendering small amounts of virus released from reservoirs inconsequential.^105^ As was the case for antiretroviral therapy, mathematical models are being used to predict how and when these therapies would work, interpret their outcomes in trials, and help guide drug development efforts (see related reviews 54, 106) (Figure 6).

### 3.1 What maintains the latent reservoir and how can we reduce or clear it?

One branch of HIV cure research is focusing on developing therapeutics that can perturb the latent reservoir, ideally reducing its size or activity such that the risk of latently infected cells reactivating and rekindling infection when ART is stopped is removed.^107^ In imagining such therapies, researchers have sought to better understand the processes that maintain a nearly stable population of latent cells despite decades of treatment and extremely low levels of detectable virus. The latent reservoir persists mainly as proviruses integrated into the genomes of infected resting memory CD4+ T cells. The frequency of these latently infected cells is around 1 per million cells^53^, ^108^, ^109^ (depending on the particular assay used and the requirement for virus functionality), and its size decays with a half‐life of 44 months on average.^49^, ^50^ The majority of evidence supports the fact this reservoir is maintained by the underlying dynamics of these cells, and not by ongoing viral replication, which could lead to continual reservoir seeding despite antiretroviral therapy (Reference 54, 110, 111). While it was originally believed by many that latently infected cells must be intrinsically long‐lived, since cell division was expected to reactivate viral expression and lead to eventual cell death, a series of studies over the past few years have convincingly demonstrated that cells in the reservoir can proliferate while remaining latently infected (Reference 110, 112). These studies have identified multiple latently infected cells — even in small samples — with virus integrated into identical sites^113^, ^114^, ^115^ in the genome or with sequence‐identical virus^116^, ^117^, ^118^, ^119^ — two findings that would be exceedingly unlikely to occur in two independent infection events and likely reflect division of infected cells.

**Figure 7:**
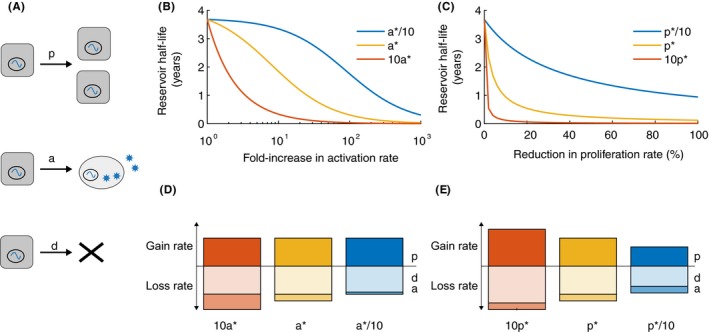
Dynamics of the latent reservoir during antiretroviral therapy in response to hypothetical treatments. (A) Diagram of the three main processes thought to impact the population of latently infected cells during therapy: cell proliferation (*p*), reactivation (*a*), and death (*d*). (B/C). Changes in the half‐life of the latent reservoir when therapies are administered that perturb one of the parameters. Calculated using Equation [Link], where half‐life = log(2)/ *δ*,* δ *= *d* + *a* − *p*. Baseline parameter values, taken from Reeves et al., are a* = 5.7× 10^−5^/d, p* = .015/d, and d* = .0155/d, *δ* = 5.2e − 4/d, half‐life = 44 months or 3.7 years (yellow lines). Red and blue lines are for alternate parameter sets. B) Hypothetical therapy that increases the activation rate (*a*) of latently infected cells during ART. When pretherapy *a* is varied (to 10a* or a*/10), *p* is kept constant at p* but *d* is adjusted to keep *δ* the same. (C) Hypothetical therapy that decreases the proliferation rate (*p*) of latently infected cells during ART. When pretherapy *p* is varied (to 10p* or p*/10), *a* is kept constant at a* but *d* is adjusted to keep *δ*. the same. (D/E) Comparison of the relative magnitude of dynamic rates for the corresponding scenarios in the figure above. The height of the bar is proportional to the log10 of the value of the rate. The bar above the horizontal axis represents the process that contributes to reservoir increase (“gain rate”, *p*) whereas bars below are processes that contribute to reservoir decay (“loss rate”, *a, d*).

The first class of drugs to be investigated to target latent infection was the so‐called “latency‐reversing agents”. The rationale for these drugs is to *increase* the rate at which HIV expression is restarted in latently infected cells. If these drugs are given along with antiretroviral therapy, then these reactivated cells will release virus but the released virus will not be able to spread infection to other cells. Eventually, the productively infected cells should die — either by viral cytopathic effects or cytotoxic immune responses.^120^ Now that the role of proliferation in maintaining the reservoir has been established, there is renewed interest in developing “antiproliferative” therapies for HIV, which would reduce the ability of latently infected cells to self‐renew. Mathematical models have been developed to predict how effective these treatment strategies are likely to be.^121^, ^122^ Two recent papers used a similar approach which we will summarize here. If it is assumed that antiretroviral therapy is 100% effective, then the viral dynamics equations (eg, Equations [Link]) become linear and much simpler, as the dynamics of the latent reservoir become uncoupled from other variables. The dynamics of the expected number of latent cells (*L*) over time can be described by

$$\dot{L}=pL-aL-d_{L}L$$

(6) $$L\left(t\right)=L_{0}e^{-\left(d_{L}+a-p\right)t}$$

where as before, *a* is the rate of latent cell reactivation, *d*_*L*_ is the rate of latent cell death, and now we have added a term *p* for proliferation (division) of these cells. To be complete, we could also track activated cells produced when latently infected cells reactivate, and the possibility for these cells to revert to latency (as in Equation [Link]), but adding these dynamics just changes the effective value of the rate *a*. This equation describes simple exponential decay of the latent pool, where the observed decay rate *δ* is determined by the sum of these rates (*δ* = *d*_*L*_ + *a* − *p*). This model can be used to examine how increases in either *a* (the target of latency‐reversing agents) or *p* (the target of antiproliferative therapy) alter the net decay rate. However, the effect of interventions on these parameters strongly depends on their underlying values, and while the observed half‐life of the reservoir tells us about *δ* (=log(2)/44 mo = .2/y), it is extremely difficult to estimate the relative contribution of each process. Petravic et al.,^122^ examined how these uncertainties effect estimates of efficacy for interventions on *a*, while Reeves et al.,^121^ explored similar questions for *p*. In Figure 7, we show examples of some output from their models.

Values for the baseline rate of reactivation of latent cells can be estimated from the timing of viral rebound,^28^, ^121^, ^123^ and in vivo cell proliferation rates can be estimated from stable isotope infusion experiments that label cells and track label decay over time.^25^, ^121^ With these estimates, *p* and *d*_*L*_ are around two orders of magnitude larger than *a*, and so large increases in *a* by a hypothetical latency‐reversing drug are predicted to be needed to significantly decrease the half‐life of the reservoir (Figure 7B, yellow line). If estimates of *a* are off and it is actually 10‐times larger, therapy outcomes are more optimistic (Figure 7B, red line). For antiproliferative therapy, even very small reductions in the latent cell division rate could dramatically increase reservoir decay (Figure 7C, yellow line), though the benefit is more modest if latent cell turnover is lower than the values measured in all central memory T cells (eg, Figure 7C, blue line).

These results highlight the difficulty of predicting therapy outcomes from models, even qualitatively, when the underlying parameters are difficult to measure. There are many reasons why cells latently infected with HIV may not have the same turnover rates as typical resting memory CD4+ T cells. For example, proviral integration may occur in host genes responsible for cell division or survival and impact their rates, a subpopulation of cells may be more susceptible to latent infection, immune killing of infected cells expressing viral proteins may select over time for cells in a “deeper” latency, or cells with particular antigen specificities may be preferentially infected and maintained over time. Preliminary trials of reservoir‐targeted drugs have had their own challenges. Latency‐reversing agents have had some success in increasing HIV gene expression but have not impacted reservoir size,^124^, ^125^, ^126^ perhaps because of their lack of specificity for the HIV promotor, posttranscriptional blocks, and lack of recognition of cells by cytotoxic immune responses. Antiproliferative therapies are still at an early stage, but it will likely be difficult to find compounds that substantially reduce division of infected cells without being overtly immune suppressive or triggering compensatory mechanisms to maintain cell population sizes.

The differential equation‐based model above can give estimates for the expected decay rate of the latent reservoir, but to achieve cure, the probability that at least one cell remaining in the reservoir reactivates and restarts high‐level infection before dying must be zero. To estimate these odds, a stochastic model is needed. An example of this type of calculation is given in Hill et al.,^123^ Like the above calculation, the exact relationship between reservoir size and probability of cure predicted from the stochastic model is highly dependent on estimates of the underlying parameter values.

### 3.2 What can viral dynamics tell us about the mechanism of action of new immunotherapies?

Another approach to treat and ideally cure HIV infection involves immunotherapies, which perturb the immune response to infection, either by boosting antiviral immune responses or reversing infection‐induced immune suppression.^127^ There are many types of immunotherapeutic agents, ranging from small‐molecules that act on immune signaling pathways, to biologics like broadly neutralizing antibodies, checkpoint inhibitors, or vaccines, to cell therapies including chimeric antigen receptor T cells. These drugs are being examined alone or in combination with ART for their ability to promote either sterilizing or functional cures for HIV. Even in the few trials that have already been conducted, mathematical models are helping to understand the mechanism of these therapies.

In recent studies by Caskey et al.,^128^ and Lu et al.,^129^ the broadly neutralizing antibody (“bNAb”) 3BNC117 was administered to previously untreated HIV+ individuals. Broadly neutralizing antibodies bind and inhibit infection by a wide range of HIV strains with high potency. Similar to trials of antiretroviral therapy, the kinetics of viral decay can be examined in the context of viral dynamics models. However, unlike antiretroviral therapy, bNAbs may alter the clearance rate of virus or the lifespan of infected cells in addition to blocking new infection events. Therefore, decay curves may be more sensitive to the efficacy of the therapy and not just the underlying pretreatment dynamics of infection. These studies found that viral load decay during 3BNC117 treatment was faster than that seen during ART, but much slower than the model predicted if the effect of the antibody was only to clear virus.^129^ However, if the model was augmented to include the ability of the antibody to mediate killing of infected cells, it could reproduce the observed kinetics. (Certain antibodies are capable of “antibody‐dependent cell‐mediated cytotoxicity” or ADCC, whereby cells bound with antibody are lysed by cytotoxic immune cells). After this novel mechanism was suggested by modeling, detailed experiments were done in human cells in culture and in humanized mice to show that indeed 3BNC117 could lead to killing of infected cells. This preliminary study only administered a single infusion of antibody, which reduced viral load by ~1.5 log before antibody washed out and infection levels increased toward baseline. It remains to be seen whether repeated long‐term treatment could lead to eventual control or whether viral rebound will always occur when the therapy is withdrawn.

**Figure 8:**
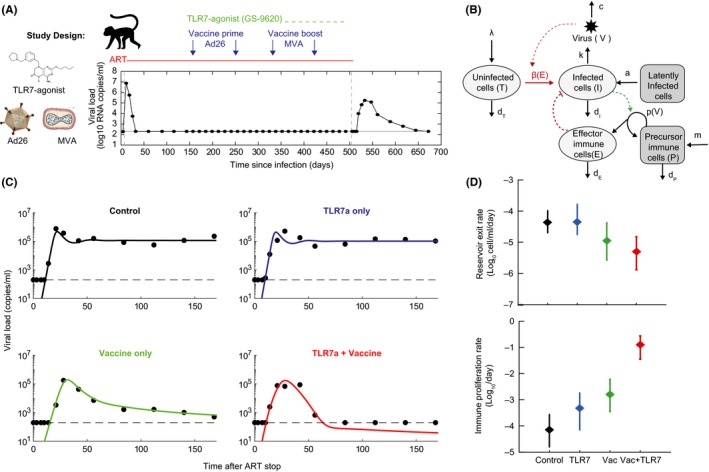
Modeling viral rebound following ART and immunotherapy. (A) Design of a study in which two novel immunotherapies, a TLR7‐agonist and a therapeutic vaccine (Ad26/MVA), where administered during ART treatment of SIV‐infected rhesus macaques, followed by a treatment interruption.^130^ The time‐course of viral loads for one example animal is shown. (B) A mathematical model of viral dynamics augmented to include an antiviral immune response that is stimulated in a viral load‐dependent way. (C) Example time‐courses of viral load for one animal from each treatment group, along with fits to the model. Each animal was fit to the model individually in a Bayesian framework (with six estimated parameters), and maximum a posteriori values for each parameter were used to plot the results. (D) Group mean values (for 8–9 animals per group) and standard deviations of two parameters that displayed significant variation between groups.

Another way of administering immunotherapy for HIV is to give it in conjunction with antiretroviral therapy. The idea is that immunotherapy could help facilitate the clearance of latently infected cells that are stochastically or that immunotherapy could help prime the immune system in the presence of low‐level antigen due to residual release from reservoirs. To test the efficacy of this strategy, all therapies are stopped, and viral load is monitored over time. When and if rebound occurs, the timing and kinetics can be used to understand the effect of treatment. Models suggest that different hypothetical treatment effects should lead to different alterations in rebound kinetics compared to the ART only case (Figure 8).

A few earlier studies conducted this type of “structured” or “analytic” treatment interruption and have provided proof‐of‐principle for using rebound as a measure of preinterruption infection status. In the AUTOVAC study, individuals on long‐term suppressive ART underwent a series of consecutive treatment interruptions.^46^ During each interruption, viral loads rebounded, and once levels passed a threshold therapy was restarted for three months before another interruption. This study found that in the second and third interruptions, the rate of exponential increase in viral load was decreased compared to the first interruption (doubling time increased from 1.4 to 1.9 days), whereas the inferred initial level of viremia from which rebound started — which is directly related to the “reservoir” size and exit rate — was higher (by ~10‐fold). These findings suggest that during later interruptions, the immune response may have been boosted compared to the first, which would be expected if the time between interruptions was short enough that some of the shorter lived antiviral immune response stimulated by the earlier interruptions persisted (long‐lived memory cells from pretreatment exist at all interruptions). They additionally suggest that the pool of cells contributing to rebound is increased at later interruptions. Although rebound after long‐term ART is generally assumed to arise from reactivated latently infected cells, it is unlikely that these short interruptions substantially increased the reservoir size compared to everything that was seeded before initial therapy.^131^, ^132^ Instead, it is more likely that the three months of treatment between interruptions was insufficient to clear intermediate‐lifespan‐infected cells.^133^ These modeling findings agree with follow‐up experimental work, which showed an increase in HIV‐specific CD8 T cells during later interruptions.^134^

A study conducted in SIV‐infected rhesus macaques, a highly representative animal model for infection that uses a virus closely related to HIV,^135^ examined the impact of the time of starting ART on later rebound.^136^ Therapy was started at a range of times between 3 days and 2 weeks after infection, and then after 6 months treatment was withdrawn. All animals experienced viral rebound, but the kinetics differed between groups. We would expect that animals starting treatment earlier would have smaller latent reservoir sizes (less opportunity for seeding) and weaker antiviral immune responses. Both experimental assays and fitting viral dynamics models to rebound trajectories supported these hypotheses: very early initiation of therapy lead to the steepest increase in viremia during rebound but the longest delay until the first detectable viral load, which are the predicted effects of lower rates of reservoir exit and decreased effective viral fitness (eg, Figure 8).

Neither very early therapy initiation or repeated treatment interruptions are effective or scalable interventions, but these studies do provide a proof‐of‐concept that viral rebound kinetics are reflective of preinterruption interventions and they have informed the analysis of two recent preclinical immunotherapy studies. The main drug of interest in these studies was an agonist of Toll‐like receptor 7 (TLR7), which is involved in the innate immune system response to viral infections. In the first study, the TLR7‐agonist was given to SIV‐infected macaques during suppressive ART, and later all treatments were stopped.^137^ Most animals rebounded in both treatment (TLR7 + ART) and control (ART only) groups, and mathematical modeling of rebound kinetics showed that rebound trajectories were altered slightly in groups receiving the TLR7 agonist in a way that suggested a partial reduction in the latent reservoir along with alterations to target cell levels and viral immune responses.^137^ Consistent with these suggestions, many animals experienced transient increases in viral load during TLR7‐agonist administration, despite ART, suggesting that this therapy had an unexpected latency‐reversing effect, and two of the thirteen animals in the intervention group never had detectable viremia after therapy cessation.

In a follow‐up study,^130^ the TLR7 agonist was tested along with a therapeutic vaccine product (both given during ART). In some animals treated with the vaccine, with or without the TLR7‐agonist, viremia rebounded rapidly to high levels but was then controlled to very low or completely undetectable levels. These dynamics are never produced by the basic viral dynamics model, which always leads to chronic infection. Alternative models were explored to explain the observations. A model that includes a population of cells belonging to the adaptive immune response which expand in response to viral antigen and act to reduce infection could explain the kinetics, and allowed for estimates of the relative contribution of reductions in the latent reservoir vs enhanced immunity in the altered kinetics.^130^ Overall, the modeling analysis suggested that the role of the vaccine was not in boosting clearance of latently infected cells prior to therapy interruption, but in creating an effective primed population of immune cells that do not exist in animals treated only with ART.

While these models have provided insight into treatment interruption trials was a way to evaluate HIV cure studies, there is significant room for improvement in future studies. A major limitation is the lack of detailed longitudinal data on levels and functionality of a panel of components of the immune response, which would allow modelers to conduct more formal hypothesis testing about potential mechanisms. The models used to explain these data are completely deterministic, whereas reactivation from latency, especially following reservoir‐reducing interventions, may be highly stochastic.^123^, ^138^ They also only track a single strain of virus, but it is possible that fitness differences between multiple strains that exit the reservoir and contribute to rebound, or that new strains that arise via mutation early in rebound contribute to viral and immunologic dynamics. For example, the number of antigenically distinct strains that reactivate may impact the chance of immune control. Another limitation is the uncertainty about the time it takes antiretroviral therapy to effectively “wash out” of the system after the last dose is taken. Hence, the relative contribute of drug washout, waiting time to latent cell reactivation, and time for infection to grow to the detection limit are hard to separate, which limits the quantitative interpretation of reservoir reactivation rates estimated from models. Closer connections between modelers and experimentalists in the early‐stage design of HIV cure trials will help ensure that mathematical model can be as informative as possible.

## 4 CONCLUSIONS

Mathematical models have been used to understand the dynamics of HIV within individual patients ever since the infection was first identified. These “viral dynamics” models have provided many important insights into infection and have been extensively used to characterize the response of HIV to antiretroviral therapy. They have elucidated the rapid turnover rate of virus‐producing cells during infection, suggested the presence of long‐lived latent cells that occasionally reactivate, and predict risks of the emergence of drug resistance during treatment. Now that research efforts are underway to find curative therapies for HIV, new models are being developed to help guide the development of these drugs, such as latency‐reversing agents, antiproliferative therapies, and immunotherapies. Models are being used to interpret the kinetics of viral rebound when antiretroviral therapy is stopped and to predict the likelihood of cure under different investigational therapies.

Despite the many successes of viral dynamics models for HIV, there are still aspects of infection that cannot be reproduced with mechanistic mathematical models, highlighting the gaps in our understanding of HIV pathogenesis. For example, a unifying explanation for the long‐term progression of infection and the development of AIDS is still lacking. HIV is a rapidly evolving infection and the population genetics of infection have been extensively characterized, but there is a general disconnect in the literature between viral dynamics models that incorporate evolution and analysis of viral sequence data. HIV dynamics are intricately connected to the population dynamics of lymphocytes, which can act as both prey and predators of the virus. However, most models of these populations are relatively heuristic and longitudinal functional data on these cell populations with which to compare models are rare. Much work remains to be done in the field of viral dynamics modeling of HIV. The development of next‐generation methods to connect mechanistic models to high‐throughput biological data, and the rapid expansion in the classes of drugs that can be used to perturb infection, have the potential to close some of these gaps in our understanding of host‐pathogen interactions for HIV.
